# Supplementary material for: Mapping global acceptance and uptake of COVID-19 vaccination: A systematic review and meta-analysis
Source: Commun Med (Lond). 2022 Sep 12;2:113. doi: 10.1038/s43856-022-00177-6 (PMC9465145; doi:10.1038/s43856-022-00177-6)
Supplement: Supplementary file 1 — Description of Additional Supplementary Files [file 43856_2022_177_MOESM1_ESM.pdf]

Description of Additional Supplementary Files

File Name: Supplementary Data 1

Description: Search strategy for studies focusing in acceptance and uptake of COVID-19 vaccination.

File Name: Supplementary Data 2

Description: Source dataset underlying all analyses, figures, and table. **Sheet 1** Total 519 included studies (>12 score): data abstraction including characteristics, study design, and primary outcomes. **Sheet 2** Acceptance and uptake of COVID-19 vaccines in each population group. **Sheet 3** Acceptance and uptake of COVID-19 vaccines across countries. **Sheet 4** Time trends of COVID-19 vaccination acceptance. **Sheet 5** Subgroup analysis of COVID-19 vaccination acceptance and uptake by sociodemographic characteristics.

File Name: Supplementary Data 3

Description: Appraisal tool for Cross-Sectional Studies (AXIS tool) used for assessing the study quality.

File Name: Supplementary Data 4

Description: The results of quality assessment of studies.

File Name: Supplementary Data 5

Description: The bibliography of all the 519 included studies.
