# Supplementary material for: Mapping global acceptance and uptake of COVID-19 vaccination: A systematic review and meta-analysis
Source: Commun Med (Lond). 2022 Sep 12;2:113. doi: 10.1038/s43856-022-00177-6 (PMC9465145; doi:10.1038/s43856-022-00177-6)
Supplement: Supplementary file 2 — Supplementary Information [file 43856_2022_177_MOESM2_ESM.pdf]

Mapping global acceptance and uptake of COVID-19 vaccination:

a systematic review and meta-analysis

Supplementary Information

AUTHORS

Qian Wang[1#], Simeng Hu[1#], Fanxing Du[1], Shujie Zang[1], Yuting Xing[1], Zhiqiang Qu[1], Xu Zhang[1], Leesa Lin[2,3], Zhiyuan Hou[1*]

#co-first authors

*Correspondence to: zyhou@fudan.edu.cn

AFFILIATIONS

[1]School of Public Health, Global Health Institute, Fudan University, Shanghai, China

[2]Department of Infectious Disease Epidemiology, London School of Hygiene & Tropical Medicine, London, United Kingdom

[3]Laboratory of Data Discovery for Health (D24H), Hong Kong Science Park, Hong Kong SAR, China

Supplementary Table 1. Sensitive analysis for COVID-19 vaccination acceptance

Population groups	Total studies			Studies excluding those with participants less than 300		
	No. of studies	No. of participants	Estimated acceptance (%, 95% CI)	No. of studies	No. of participants	Estimated unwillingness (%, 95% CI)
Overall	476	7967690	67.8 (67.1-68.6)	430	7949605	67.6 (66.8-68.5)
Adults	202	7068345	69.1 (68.2-70.1)	193	7064238	69.1 (68.1-70.1)
Healthcare workers	98	129265	67.5 (64.4-70.6)	84	124017	66.1 (62.5-69.6)
Patients with chronic diseases	52	166278	67.4 (63.9-70.9)	41	163304	65.8 (61.9-69.7)
Pregnant/breastfeeding women	13	25102	54.0 (46.3-61.7)	11	24298	53.2 (44.6-61.8)
University students	45	50471	67.7 (62.7-72.8)	41	49395	68.5 (63.2-73.8)
Children and adolescents	46	363825	70.7 (67.6-73.9)	42	362504	70.7 (67.4-73.9)
Others	58	164404	65.9 (60.8-71.0)	45	161849	66.3 (60.4-72.2)

For each pooled estimate, heterogeneity tests between studies reached Higgins' I^2 statistic >99%, P<0.001.
