# Supplementary material for: Mapping global acceptance and uptake of COVID-19 vaccination: A systematic review and meta-analysis
Source: Commun Med (Lond). 2022 Sep 12;2:113. doi: 10.1038/s43856-022-00177-6 (PMC9465145; doi:10.1038/s43856-022-00177-6)
Supplement: Supplementary file 9 — Reporting Summary [file 43856_2022_177_MOESM9_ESM.pdf]

nature research

Corresponding author(s): Zhiyuan Hou
Last updated by author(s): Aug 18, 2022

Reporting Summary

Nature Research wishes to improve the reproducibility of the work that we publish. This form provides structure for consistency and transparency in reporting. For further information on Nature Research policies, see our Editorial Policies and the Editorial Policy Checklist.

Statistics

For all statistical analyses, confirm that the following items are present in the figure legend, table legend, main text, or Methods section.

n/a	Confirmed	
☒	☐	The exact sample size (n) for each experimental group/condition, given as a discrete number and unit of measurement
☒	☐	A statement on whether measurements were taken from distinct samples or whether the same sample was measured repeatedly
☒	☐	The statistical test(s) used AND whether they are one- or two-sided<br>*Only common tests should be described solely by name; describe more complex techniques in the Methods section.*
☒	☐	A description of all covariates tested
☒	☐	A description of any assumptions or corrections, such as tests of normality and adjustment for multiple comparisons
☐	☒	A full description of the statistical parameters including central tendency (e.g. means) or other basic estimates (e.g. regression coefficient) AND variation (e.g. standard deviation) or associated estimates of uncertainty (e.g. confidence intervals)
☒	☐	For null hypothesis testing, the test statistic (e.g. F, t, r) with confidence intervals, effect sizes, degrees of freedom and P value noted<br>*Give P values as exact values whenever suitable.*
☒	☐	For Bayesian analysis, information on the choice of priors and Markov chain Monte Carlo settings
☐	☒	For hierarchical and complex designs, identification of the appropriate level for tests and full reporting of outcomes
☒	☐	Estimates of effect sizes (e.g. Cohen's d, Pearson's r), indicating how they were calculated

Our web collection on statistics for biologists contains articles on many of the points above.

Software and code

Policy information about availability of computer code

Data collection	Not applicable.
Data analysis	Not applicable.

For manuscripts utilizing custom algorithms or software that are central to the research but not yet described in published literature, software must be made available to editors and reviewers. We strongly encourage code deposition in a community repository (e.g. GitHub). See the Nature Research guidelines for submitting code & software for further information.

Data

Policy information about availability of data

All manuscripts must include a data availability statement. This statement should provide the following information, where applicable:

- Accession codes, unique identifiers, or web links for publicly available datasets
- A list of figures that have associated raw data
- A description of any restrictions on data availability

Data availability
All data generated or analyzed during this study are included in this article and its supplementary files. Source data for the main figures of the manuscript is available as Supplementary Data 2.

Field-specific reporting

Please select the one below that is the best fit for your research. If you are not sure, read the appropriate sections before making your selection.

☐ Life sciences ☒ Behavioural & social sciences ☐ Ecological, evolutionary & environmental sciences

For a reference copy of the document with all sections, see nature.com/documents/nr-reporting-summary-flat.pdf

Behavioural & social sciences study design

All studies must disclose on these points even when the disclosure is negative.

Study description	systematic review and meta-analysis
Research sample	We searched four peer-reviewed databases (PubMed, EMBASE, Web of Science, and EBSCO)for papers published in English from December 1, 2019 to February 27, 2022. This review included original survey studies which investigated acceptance or uptake of COVID-19 vaccination.
Sampling strategy	We employed the following search terms on four peer-reviewed databases (PubMed, EMBASE, Web of Science, and EBSCO) and three preprint databases (medRxiv, Europe PMC, and SSRN): coronavirus terms ("coronavirus disease" OR coronavirus OR coronaviruses OR 2019-nCoV OR 2019ncov OR COVID-19 OR "severe acute respiratory syndrome coronavirus 2" OR SARS-2 OR SARS-COV-2) AND vaccine terms (vaccin* OR immunis* OR immuniz*) AND survey terms (survey OR questionnaire OR poll). All papers published in English from December 1, 2019 to February 27, 2022 were collected with the above search terms. Among 15690 identified studies, 519 articles with 7,990,117 participants were eligible for inclusion.
Data collection	Two researchers independently performed article extraction. When inconsistency arose, they were asked to discuss and revisit the article until reaching a consensus. We extracted the following information from the included articles: title, first author, publication date, journal, study design, sampling method, sample size, survey period, survey location, target population, and measurement questions about COVID-19 vaccination acceptance. To achieve the study objectives, we also extracted four outcomes: (1) overall acceptance of COVID-19 vaccination (total sample); (2) subgroups' acceptance of COVID-19 vaccination (by gender, age, race, education, and income); (3) overall uptake of COVID-19 vaccination (total sample); and (4) subgroups' uptake of COVID-19 vaccination (by gender, age, race, education, and income).
Timing	We collected related papers published from December 1, 2019 to February 27, 2022.
Data exclusions	Articles were included in this review if they investigated acceptance, willingness, intention, or uptake of COVID-19 vaccination, and if they were original survey studies. We excluded studies that investigated (1) non-COVID-19, clinical-trial, emergency or boosting vaccination acceptance, (2) studies which assessed willingness-to-pay or conditional acceptance, (3) studies without outcomes of interest, or (4) studies that applied continuous variables to evaluate vaccination acceptance. Studies using continuous variables usually adopted different response ranges with no consistent meanings for response options across studies, and there were also no clear cut-off points for vaccination acceptance or refusal in continuous variables. Therefore, studies with continuous variables were excluded in our review for conducting the meta-analysis of vaccination acceptance rate. The following study designs were also excluded: editorials, letters, commentaries, correspondences, study protocols, reviews, qualitative studies, intervention studies, and non-survey studies such as crawling information from social media. Two independent researchers first screened titles and abstracts, and then scrutinized the full texts to estimate their eligibility. When they had disagreements on study selection, the third researcher was consulted.<br>A total of 15690 articles were identified through database searches. After excluding 8073 duplicates and a further 6124 from the screening of titles and abstracts, 1493 articles were full-text screened for eligibility. Ultimately 519 articles were eligible for inclusion, with 7,990,117 participants included in the meta-analysis.
Non-participation	Not applicable.
Randomization	The pooled acceptance rate of COVID-19 vaccination was estimated by different populations, countries, survey times, and participants' characteristics. We categorized all study participants into seven groups: (1) adults, (2) healthcare workers, (3) patients with chronic diseases, (4) pregnant or breastfeeding women, (5) university students, (6) children and adolescents, and (7) other populations. Other populations were defined as study populations that cannot be categorized into the first six study populations. We first estimated the pooled acceptance or uptake of COVID-19 vaccination for each population group, and within each population group, we then estimated the acceptance of COVID-19 vaccination in individual countries by synthesizing all studies from the same country. To compare the trends of COVID-19 vaccination acceptance over time, we reported the pooled acceptance rate of all studies from the same survey month, and developed graphs to illustrate the time trends. For acceptance estimates from adults, we also conducted subgroup analyses based on their sociodemographic characteristics such as gender, age group, race, education, and income.

Reporting for specific materials, systems and methods

We require information from authors about some types of materials, experimental systems and methods used in many studies. Here, indicate whether each material, system or method listed is relevant to your study. If you are not sure if a list item applies to your research, read the appropriate section before selecting a response.

Materials & experimental systems

n/a	Involved in the study
☒	☐ Antibodies
☒	☐ Eukaryotic cell lines
☒	☐ Palaeontology and archaeology
☒	☐ Animals and other organisms
☒	☐ Human research participants
☒	☐ Clinical data
☒	☐ Dual use research of concern

Methods

n/a	Involved in the study
☒	☐ ChIP-seq
☒	☐ Flow cytometry
☒	☐ MRI-based neuroimaging
